# Bacterial activity in cystic fibrosis lung infections

**DOI:** 10.1186/1465-9921-6-49

**Published:** 2005-06-01

**Authors:** Geraint B Rogers, Mary P Carroll, David J Serisier, Peter M Hockey, Valia Kehagia, Graeme R Jones, Kenneth D Bruce

**Affiliations:** 1 Department of Life Sciences, King's College London, London, UK; 2 Cystic Fibrosis Unit, Southampton University Hospitals NHS Trust, UK; 3 Health Protection Agency, Southampton Laboratory, UK; 4 The Adult Cystic Fibrosis Unit, Mater Adult Hospital, Brisbane, Australia

**Keywords:** Cystic Fibrosis, bacterial infections, 16S rDNA, T-RFLP profiling, bacterial activity

## Abstract

**Background:**

Chronic lung infections are the primary cause of morbidity and mortality in Cystic Fibrosis (CF) patients. Recent molecular biological based studies have identified a surprisingly wide range of hitherto unreported bacterial species in the lungs of CF patients. The aim of this study was to determine whether the species present were active and, as such, worthy of further investigation as potential pathogens.

**Methods:**

Terminal Restriction Fragment Length Polymorphism (T-RFLP) profiles were generated from PCR products amplified from 16S rDNA and Reverse Transcription Terminal Restriction Fragment Length Polymorphism (RT-T-RFLP) profiles, a marker of metabolic activity, were generated from PCR products amplified from 16S rRNA, both extracted from the same CF sputum sample. To test the level of activity of these bacteria, T-RFLP profiles were compared to RT-T-RFLP profiles.

**Results:**

Samples from 17 individuals were studied. Parallel analyses identified a total of 706 individual T-RF and RT-T-RF bands in this sample set. 323 bands were detected by T-RFLP and 383 bands were detected by RT-T-RFLP (statistically significant; P ≤ 0.001). For the group as a whole, 145 bands were detected in a T-RFLP profile alone, suggesting metabolically inactive bacteria. 205 bands were detected in an RT-T-RFLP profile alone and 178 bands were detected in both, suggesting a significant degree of metabolic activity. Although *Pseudomonas aeruginosa *was present and active in many patients, a low occurrence of other species traditionally considered to be key CF pathogens was detected. T-RFLP profiles obtained for induced sputum samples provided by healthy individuals without CF formed a separate cluster indicating a low level of similarity to those from CF patients.

**Conclusion:**

These results indicate that a high proportion of the bacterial species detected in the sputum from all of the CF patients in the study are active. The widespread activity of bacterial species in these samples emphasizes the potential importance of these previously unrecognized species within the CF lung.

## Introduction

The Microbiological analysis of clinical specimens has relied traditionally on cultivation prior to identification. However, recent genetic innovations promise to provide dramatic advances in diagnosis and fresh insights into infections that have previously been considered well characterised. The use of molecular biological approaches in clinical scenarios obviates the requirement for *in vitro *culture prior to analysis and so removes problems associated with microbial cultivation. In addition, these approaches are ideal for cases such as trauma where predicting the pathogen(s) responsible is challenging.

Molecular biological approaches have gained widespread acceptance for the study of bacterial communities in natural environments [reviewed in [1-3]]. Here, nucleic acids, extracted directly from samples, act as templates for PCR amplification of phylogenetically-informative ribosomal sequences using oligonucleotide primers "universal" for the Domain Bacteria. This means that no prior assumptions are made about the identity of species present. Bacterial community composition can then be assessed through sequence analysis of cloned ribosomal PCR products and by Terminal Restriction Fragment Length Polymorphism (T-RFLP) profiling [4,5].

Here, we focus on bacterial infections within the lungs of cystic fibrosis (CF) patients. There are more than 5,000 registered CF patients in the UK [6]. Although CF patient life expectancy has steadily increased, the mortality rate for patients aged between 26 and 30 years remains at around 50 per 1000 per year [7]. Mortality is primarily determined by repeated infective exacerbations. Ultimately, 80 to 95% of CF patients succumb to respiratory failure brought on by chronic bacterial infection and concomitant airway inflammation [8]. The characterisation of the bacteria present in the CF lung is critical if therapy is to be advanced. Moreover, we suggest that this strategy can benefit a diverse range of clinical scenarios.

Previous molecular biological studies have shown that the level of bacterial diversity in adult CF sputum was much higher than previously recognised [9-12] and that the communities detected were distinct. This contrasts sharply with wisdom informed by traditional screening of sputa that focuses on only a few pathogenic species including *Pseudomonas aeruginosa*, *Staphylococcus aureus *and the *Burkholderia cepacia *complex.

Moreover, many of the species detected were anaerobes – often obligate – from within the genera *Bacteroides*, *Eubacterium*, *Fusobacterium*, *Porphyromonas*, *Prevotella*, *Rothia *and *Veillonella*. This agreed with earlier studies [13,14] and may be of particular relevance given the increased recognition of the importance of anaerobic growth in CF infections [15]. In addition, many of the species identified, including *Abiotrophia adiacens*, *Mycoplasma salivarium*, *Ralstonia taiwanensis*, *Rothia mucilaginosus *and *Staphylococcus hominis*, had not been reported as previously isolated from CF sputum. Although the role of these species in lung pathogenesis has yet to be determined, the first step is to establish whether they are active within the CF lung. Although activity does not necessarily imply pathogenicity, it strongly suggests that further study is warranted.

Here, we assess the extent to which bacteria in sputum sampled from the CF lung were active by exploiting the difference in stability of DNA and RNA. As 16S rRNA is inherently unstable, it can be used to define intact, metabolically active bacterial cells [16] with bacterial metabolic activity inferred from the level of transcription of these sequences [17]. Reverse Transcription Terminal Restriction Fragment Length Polymorphism (RT-T-RFLP), performed in parallel with T-RFLP provides an accepted means of determining relative metabolic activity within communities [18]. T-RFLP and RT-T-RFLP analyse the same genetic sequence. The single difference is that in RT-T-RFLP, complementary DNA (cDNA) copies, generated from 16S rRNA, are used as the template instead of 16S rDNA. Here, we wished to test the hypothesis that the majority of the species identified in the CF lung are active. To do this, two profiling approaches were used to study DNA and RNA extracted directly from the same clinical sample taken from 17 CF patients. Further, the T-RFLP profiles generated from the 17 CF sputa were compared to those generated from sputa obtained from 19 healthy, non-CF individuals.

## Materials and methods

### Clinical samples and preparation for nucleic acid extraction

Sputum samples were collected from 17 adult CF patients attending Southampton University Hospital, with ethical approval granted by the Southampton Research Ethics Committee (067/01). 12 patients were suffering infective exacerbation at time of sampling, whilst five were considered to be stable. Ten volumes of RNA*later *solution (Promega, Southampton, UK) were added to sputum samples immediately following collection following manufacturer's instructions. Prior to nucleic acid extraction, samples were prepared for nucleic acid extraction, using a series of washes and sputasol treatment as described previously [10].

### DNA and RNA extraction

All reagents, glassware and plastics used in RNA work were DEPC-treated prior to use. RNA was extracted as follows: 0.75 ml of Tri Reagent (Sigma-Aldrich, Dorset, UK) were added to approximately 0.2 ml of each sample and vortexed for 1 min. Samples were incubated at room temperature for 5 min prior to the addition of 0.2 ml chloroform. Samples were vortexed for 15 sec. and incubated at room temperature for 5 min. Phases were separated by centrifugation at 12,000 × *g *for 15 min at 4°C.

#### i) DNA extraction

0.3 ml of 100% ethanol was added to precipitate the DNA from the lower phase. The sample was mixed by inversion, incubated at room temperature for 3 min and centrifuged at 12,000 × *g *for 5 min at 4°C. The pellet was washed in 0.1 M sodium citrate, 10% ethanol solution (during each wash the pellet was allowed to stand for at least 30 min). Pellets were centrifuged at 12,000 × *g *for 5 min at 4°C and washed twice in 75% ethanol. The DNA was vacuum dried, with the pellet resuspended in 100 μl H_2_O and stored at -20°C.

#### ii) RNA extraction

The upper phase was transferred to a fresh microfuge tube and 0.5 ml of propan-2-ol were added. Samples were incubated for 10 min at room temperature and RNA was pelleted by centrifugation at 12,000 × *g *for 10 min at 4°C. The supernatant was removed and the RNA pellet washed once in 75% ethanol and re-pelleted by centrifugation at 7,500 × g for 5 min at 4°C. Pellets were air-dried for 10 min, resuspended in 30 μl distilled water and incubated for 10 mins at 55°C. Purified RNA samples were stored as aliquots at -70°C.

Prior to reverse transcription, any residual DNA was removed using DNAseI (Epicentre, Madison, USA) in accordance with the manufacturer's instructions, with PCR amplification controls performed as appropriate.

### Reverse transcription

Two universal bacterial primers were used, namely; 8f700 (5'-AGA GTT TGA TCC TGG CTC AG-3') and 920r (5'-CCG TCA ATT CAT TTG AGT TT-3') [5,10]. cDNA was generated from the isolated RNA using 920r and AMV reverse transcriptase (Promega, Southampton, UK) in accordance with the manufacturer's instructions. Double stranded DNA was generated using 1 μl of this cDNA as template in a 50 μl PCR reaction containing both primers (8f700 and 920r). PCR products amplified were verified by Tris-acetate-EDTA (TAE)-agarose gel electrophoresis on 0.8% (wt/vol) TAE-agarose gels stained in ethidium bromide (0.5 mg/L) with images, viewed on a UV transilluminator (Herolab, Wiesloch, Germany), captured by using a Herolab image analyzer with E.A.S.Y STOP win 32 software (Herolab).

### DNA quantification

Extracted DNA and restricted PCR products (below) were quantified using a CytoFluor series 4000 multiwell plate reader (PerSeptive Biosystems, Foster City, USA) using the PicoGreen DS DNA quantitation kit (Molecular Probes, Lieden, Netherlands) following the manufacturer's instructions.

### T-RFLP amplification and profiling

PCR products for T-RFLP analysis were amplified using primers 8f700 (labelled at the 5'end with IRD700) and 926r from *c*. 20 ng of extracted DNA as previously described [10]. PCR products (*c*. 20 ng) were digested to completion using 1 U of the restriction endonuclease *Cfo*I, with *c*. 0.7 μg of T-RFLP PCR products were separated by length using a LI-COR IR2 automated DNA sequencer again as previously described [10]. The gels were analysed by using GeneimageIR v.3.56 (Scanalytics, Fairfax, USA). When profile data were assessed, only peaks of ≥ 0.1% of the total lane signal were classified as bands for further analysis. The positions of these individual bands were calculated in relation to microSTEP 15 a (700-nm) size marker (Microzone, Lewes, UK). A threshold of ×2 was used as a means of identifying marked differences between T-RFLP and RT-T-RFLP band volumes.

### Band Quantification and calling

The IR signal level produced by each band was determined using Phoretix 1D Advanced v.5.10, (Nonlinear Dynamics, Newcastle upon Tyne, UK). Due to the low volumes loaded, small errors could have lead to significant variations in band intensity. To avoid such errors, band volumes were determined as a percentage of the total band volume detected in each profile.

### Sputum from non-CF individuals

Sputum production was induced in 19 healthy, non-CF individuals by the inhalation of nebulised saline for 5 min. Following nebulisation, subjects were asked to rinse their mouths thoroughly with water and blow their noses. Expectorated sputum was then collected and subjected to the same DNA extraction, amplification and profiling protocols employed for CF sputum analysis. Individuals were selected at random to provide sputum samples. Individuals that reported either acute or chronic respiratory problems were excluded.

### *in silico *sequence analysis

Published bacterial 16S rRNA gene sequence data, stored at GenBank , were retrieved. MapSort (Wisconsin Package version 10.3; Accelrys) was used to predict the band sizes for T-RFLP analysis. Mapsort, which locates the position of restriction endonuclease recognition motifs in a given sequence, was used to determine the length (in bases) from the 5' end of primer 8f-700IR (see below) to the first cleavage position of the restriction endonuclease *Cfo*I in each 16S rRNA gene sequence. This process was performed on all of the bacterial entries in the Genbank database that spanned the amplified region. In this way, it was possible to predict the length of T-RF bands generated from 853 separate phylotypes (data not shown).

### Statistical analysis

For each of the bands that were detected in a T-RFLP or RT-T-RFLP profile unaccompanied by a band in the corresponding RT-T-RFLP or T-RFLP profile, the frequency of unaccompanied detection was compared with the frequency of accompanied detection in the sample set as a whole. The ratio of unaccompanied detection to total detection was multiplied by unaccompanied detection. This was performed because relatively frequently detected bands, which were unaccompanied in a majority of instances, would otherwise appear less significant. A score ≥ 2 was used as a threshold for the identification of bands that differed notably between their detection by the two techniques.

Hierarchical cluster analysis, with the Dice measure and Chi-square test using Yates correction (SPSS for Windows v.10.1, SPSS Inc., Chicago, USA) was used to construct a dendrogram representing level of similarity between the 34 bacterial community T-RFLP and RT-T-RFLP profiles studied here. Further, this process was used to compare the similarity of T-RFLP profiles generated from CF sputa with those generated from healthy, non-CF sputum.

### Role of the funding source

The sources of funding of this study were not involved in experimental design and interpretation. No influence was exerted on the decision to publish.

## Results

**Figure 1:**
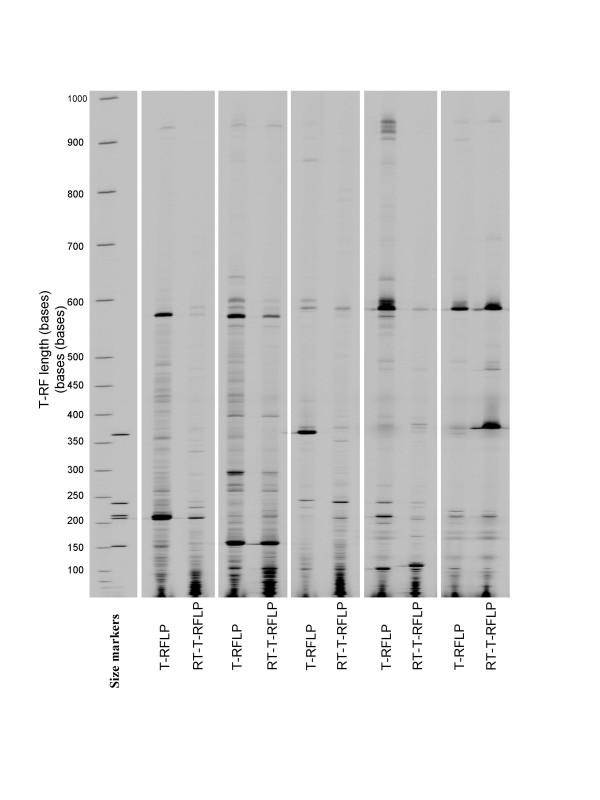
**Electrophoretic gel images generated by T-RFLP and RT-T-RFLP**. This figure shows the profiles generated from five sputum samples within the sample set. By a process of automated comparison of band positions with those in marker lanes allows their length to be determined and direct comparisons to be made between lanes.

**Figure 2:**
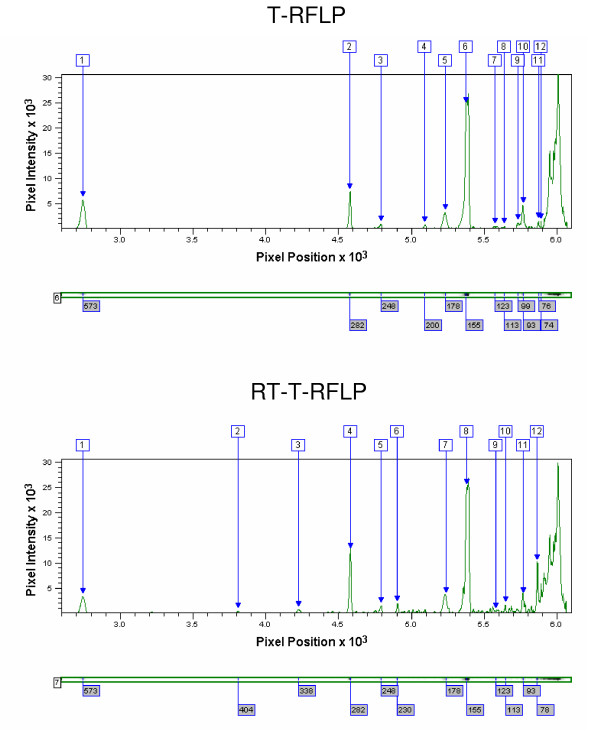
**Identification of individual bands within regions of corresponding T-RFLP and RT-T-RFLP profiles**. This figure shows regions of profiles as analysed using Phoretix 1D Advanced v.5.10 (Nonlinear Dynamics, Newcastle upon Tyne, UK). In each case, the region of electrophoretic profile is shown (below) next to a trace of relative band intensity. The manual confirmation of correct band identification minimises the inclusion of erroneous peaks.

Electrophoretic gel images generated by T-RFLP and RT-T-RFLP profiling from five sputum samples are shown in Figure 1. An example of the identification of individual bands within a region of electrophoretic profile is shown for corresponding areas of a T-RFLP and RT-T-RFLP profiles in Figure 2.

### T-RFLP and RT-T-RFLP profiling

Parallel T-RFLP and RT-T-RFLP analysis was performed on 17 sputum samples. A total of 706 individual T-RF and RT-T-RF bands were detected in this sample set. Of these, 323 were detected by T-RFLP analysis and 383 were detected by RT-T-RFLP analysis. This difference in the number of bands detected was significant (P ≤ 0.001, Chi-square test, Yates correction). The number of bands detected in profiles generated from each individual sample is shown in Table 1.

**Table 1:** Number of bands detected in T-RFLP and RT-T-RFLP profiles generated from the sample set.

**Patient**	**T-RFLP bands**	**RT-T-RFLP bands**
1	42	33
2	35	38
3	14	22
4	25	27
5	38	18
6	26	15
7	16	21
8	13	15
9	14	38
10	10	11
11	7	18
12	15	15
13	12	11
14	17	29
15	16	20
16	10	38
17	13	14
**Total**	**323**	**383**
**Average**	**19.0 (± 10.4)**	**22.5 (± 9.5)**

The number of T-RF bands detected above a threshold of 0.1% of the total lane signal volume is shown for both the T-RFLP and RT-T-RFLP profiles generated from each of the 17 samples. Standard deviations for average values are shown in brackets.

The banding positions generated through T-RFLP and RT-T-RFLP analysis were compared for each sample. 178 bands were detected in both a T-RFLP profile and the corresponding RT-T-RFLP profile (356 bands in total), 145 bands were detected in a T-RFLP profile but were absent in the corresponding RT-T-RFLP profile, and 205 bands were detected in an RT-T-RFLP profile but were absent in the corresponding T-RFLP profile.

Where a band of a given length was detected in profiles generated in a sample, it was present in both T-RFLP and RT-T-RFLP profiles in 33.7% of instances. In 27.4% of instances in was detected in the T-RFLP profile alone and in 38.8% of instances it was detected in the RT-T-RFLP profile alone.

The ratio of unaccompanied bands to total bands detected was multiplied by the number of unaccompanied bands detected. This process identified 7 band lengths with a score of ≥ 2 in T-RFLP profiles, compared with 25 band lengths in RT-T-RFLP profiles. The only band from either group whose length corresponded to that of a recognised CF pathogen was 209 bases (*B. cepacia *complex) had a score of 2.0 in RT-T-RFLP profiles. No pattern was discerned between any of the other bacterial species whose predicted T-RF length corresponded with these bands. No band length had a score ≥ 2 in both T-RFLP and RT-T-RFLP profiles.

The intensity of each of the T-RF bands detected was determined and placed in rank order (descending band volume) (Table 2, see additional file). The five highest rank ordered bands represented 39.5% (± 21.1), 14.4% (± 6.2), 8.6% (± 2.6), 5.8% (± 1.6), and 4.5% (± 2.0) of the total band signal in T-RFLP profiles respectively. For RT-T-RFLP profiles, the top five rank ordered T-RF bands represented 35.2% (± 19.4), 14.4% (± 6.9), 9.2% (± 3.3), 7.0% (± 2.9) and 4.7% (± 1.7), respectively.

Fifty five of the T-RF bands lengths detected by T-RFLP profiling were in the five most intense bands in one or more profile, compared with 53 T-RF band lengths in RT-T-RFLP profiling. T-RF bands of a given length were detected within the top five rank ordered positions of intensity in an average of 1.5 T-RFLP profiles and 1.6 RT-T-RFLP profiles. Of the T-RF band lengths identified in this way, 26 were detected in the top five rank positions in T-RFLP profiles but not RT-T-RFLP profiles, whereas 25 were detected in the top five rank positions in RT-T-RFLP profiles but not in T-RFLP profiles.

In both cases, a T-RF band of 155 bases, corresponding to that produced by *P. aeruginosa*, was the most frequently detected band in the top five rank ordered positions, being detected in 8.2% and 9.4 % of T-RFLP and RT-T-RFLP profiles respectively. The second most commonly detected T-RF band length was also the same in both profiling approaches – a T-RF band of 78 bases in length was detected in one of top five rank ordered positions in 5.9% and 4.7% of T-RFLP and RT-T-RFLP profiles respectively. Computer-based band length predictions made using published sequence data indicate that a 78 base T-RF band would be consistent with that produced by *Ectothiorhodospira mobilis*, *Methylobacter psychrophilus*, *Methylomicrobium agile*, *Methylomonas rfodinarum *and *Methylomonas rubra*.

The prevalence of other bacterial species that have been considered traditionally to be key CF pathogens was determined. In general, it was found that these were not highly represented in either the T-RFLP or RT-T-RFLP profiles. A band corresponding to *B. cepacia *complex was detected in one T-RFLP profile (patient 7), and one RT-T-RFLP profile (patient 2). These bands represented 2.2% and 3.3% of total band volume respectively. A band corresponding to *H. influenzae*, representing 1.7% of the total band volume, was detected in a single T-RFLP profile (patient 15). A band consistent with that produced by *S. maltophilia *was detected in the T-RFLP profiles generated from two patients (patients 1 and 2), representing 1.6% and 5.5% of the total band volume respectively. A band was also detected in the RT-T-RFLP profile from a third sample (patient 5), where it represented 18% of the total band volume. No band of a length corresponding to *S. aureus *was detected.

### Hierarchical cluster analysis

**Figure 3:**
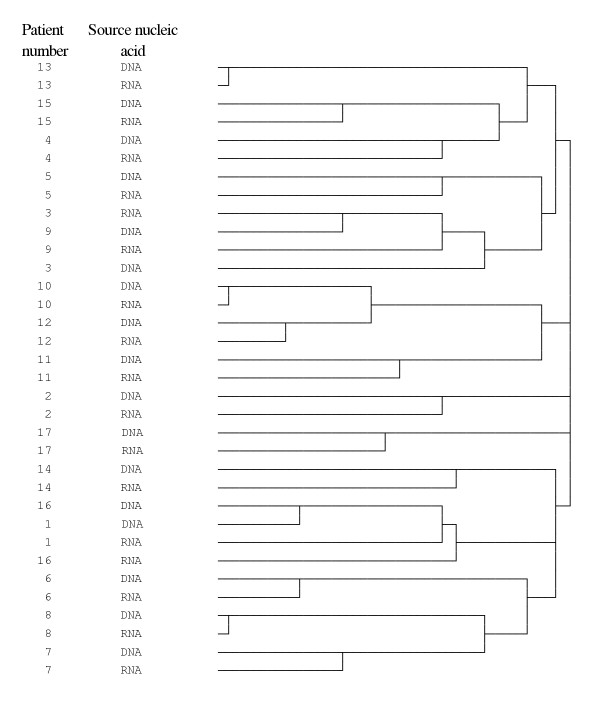
**Dendrogram constructed using T-RFLP and RT-T-RFLP profiles generated from the sample set**. A dendrogram was constructed using the results of Hierarchical Cluster Analysis (HCA), using Dice measure, of the T-RFLP and RT-T-RFLP profile data. HCA results in the formation of clusters in which profiles are iteratively joined in a descending order of similarity.

A dendrogram was derived by hierarchical cluster analysis using the Dice measure for all of the T-RFLP and RT-T-RFLP generated profiles (Figure 3). This showed that for the majority (13 of 17) patients studied, the T-RFLP and RT-T-RFLP profiles from the same sample clustered more closely than any other profile. However, profiles were observed in two instances that more closely matched those of other individuals (patients 3 and 9 and patients 1 and 16, Figure 3).

### Healthy, non-CF samples

Between 18 and 54 individual T-RF bands were detected in the T-RFLP profiles generated from the healthy, non CF sputa. On average 29 (± 10) T-RF bands were detected in each profile.

A total of 556 individual T-RF bands were resolved in the 19 samples analysed. These represented 210 different T-RF band lengths. T-RF bands of a given length were detected in between 1 and 18 of the 19 samples, being detected in 2.65 samples (± 3.67) on average. Of these 210 T-RF band lengths, 81 were detected in both the CF and non CF sputum profiles, 129 were detected in the healthy sample set only, and 114 were detected in the CF only.

Profiles generated from healthy sputum were more similar than profiles generated from CF sputum, with a greater level of overlap between the T-RF bands lengths detected in the profiles generated. In the CF sample set, no T-RF band length was detected in more than 41.1 % of profiles, with 39% of T-RF band lengths detected in a single profile only, and 22.2% of T-RF band lengths detected in two profiles, only. By comparison, only 21.9% and 12.2% of T-RF band lengths were detected in one or two healthy sputum profiles respectively. Further, 14 different T-RF band lengths were detected in 50% or more of healthy profiles and 4 were detected in more that 75% of healthy profiles.

Of the 14 T-RF band lengths detected in 50% or more of healthy sample profiles, 5 were not detected in the CF sample set at all, 3 were detected in a single sample only, two were detected in two profiles, two in three profiles, one in four profiles and one in 6 profiles.

None of the profiles generated from healthy sputa were found to contain T-RF bands of lengths corresponding to the recognised CF pathogens *P. aeruginosa, B. cepacia *complex, *S. aureus*, or *H. influenzae*. A T-RF band of 214 bases was resolved in 5 of the 19 healthy profiles. This is consistent with the T-RF band produced by both five different species (*Stenotrophomonas maltophilia*, *Fusobacterium gonidoformans*, *Aeromonas hydrophila*, *Shewanella alga*, *Vibrio wodanis*), of which *Stenotrophomonas maltophilia *is a recognised CF pathogen.

Of the 209 band lengths detected in the RT-T-RFLP profiles, 118 (56.4%) were not detected in the profiles generated from the healthy sputum sample set whatsoever.

**Figure 4:**
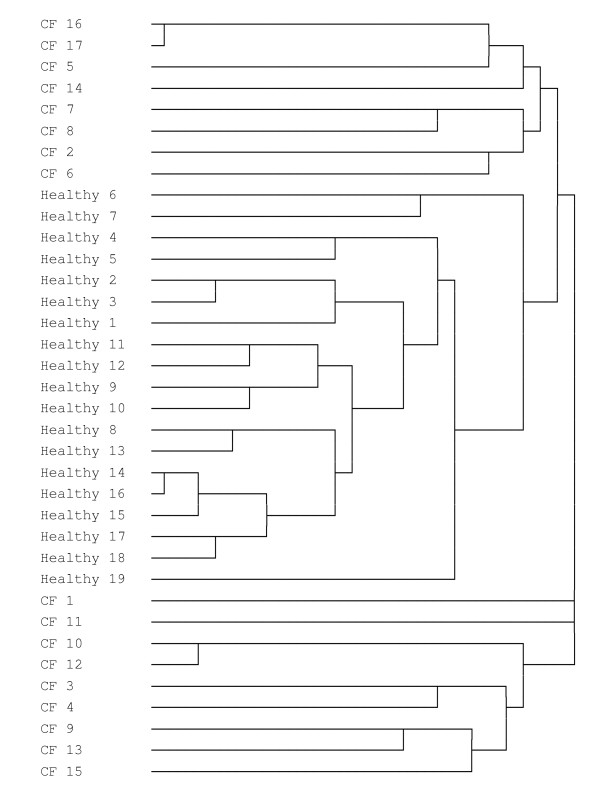
**Dendrogram constructed using T-RFLP profiles generated from sputum samples obtained from CF patients and healthy individuals**. A dendrogram was constructed using the results of Hierarchical Cluster Analysis (HCA), using Dice measure, of the T-RFLP profile data.

Hierarchical cluster analysis, using Dice similarity measure, was performed on the T-RFLP profiles generated from CF and non-CF samples. The dendrogram that was generated is shown in Figure 4. It was found that there was complete separation of cluster groupings between CF and non-CF samples.

## Discussion

This study addresses important questions about the activity of bacteria in infections. Recently, it has been shown that many bacterial species not previously associated with CF lung infections, could be detected when molecular biological approaches were applied to the study of sputa [10,19]. These studies also showed that many species in CF sputum were facultative or obligate anaerobes. This study shows that the majority of these species were metabolically active. Potentially, this has important implications for treatment and it is now critical to determine what impact these species have on lung disease and to identify their clinical significance.

The application to clinical studies of the combined approaches used here is novel. These approaches are robust and have previously been shown to be highly reproducible [data not shown, [10,12]]. Here it was found that, on average, each patient had more than 22 metabolically active bacterial species per sputum sample. This represented a statistically significant difference over species number identified through T-RFLP alone (P ≤ 0.001). This finding makes it important to determine the role of metabolically active bacteria in lung pathogenesis. Some species may emerge as frank pathogens. However, even active bacterial species that might be considered "avirulent" may play an important indirect role; for example, it has been demonstrated that avirulent oropharyngeal flora can cause an upregulation of virulence genes and consequentially pathogenicity of *P. aeruginosa *[20].

In certain cases, the T-RFLP profile and RT-T-RFLP profile were found by visual comparison to be similar. To provide a more robust analysis, Hierarchical Cluster Analysis (HCA) was used. HCA demonstrated that there was typically greater similarity between profiles generated from individual samples than any other individual. This implies that each patient has an individual collection of typically active bacteria. The lack of HCA clustering according to technique – i.e. discrete groups of T-RFLP profiles and RT-T-RFLP profiles – suggests that the same groups of species are not either "present" or "active" in different individuals. This reinforces the requirement for management to be highly specific for each individual patient, a concept that up to now has been put into practice based on clinical experience without particular scientific backing. Moreover, it may explain the differential response of patients, at apparently similar stages of CF lung disease, to antibiotic regimes.

Marked differences in T-RFLP and RT-T-RFLP profiles were however observed in many cases. For example, in 39.6% of banding positions, a signal was detected in the RT-T-RFLP profile, but not in the corresponding T-RFLP profile. This was not artefactual – there was no significant difference in the overall distribution of the total lane volume in the profiles generated by T-RFLP and RT-T-RFLP profiling. In the case of a signal not being detected in the corresponding T-RFLP profile, it is likely that cells of an individual species were present in low numbers, but exhibited very high metabolic rates. When assessing relative levels of metabolic activity, it should be noted the number of ribosomal gene operons in different bacterial species varies. For example, *Rickettsia prowazekii *and *Mycoplasma pneumoniae *have only one ribosomal operon [21,22], whereas *Clostridium paradoxum *has 15 ribosomal operons [23]. Therefore, in a diverse bacterial community such as is present in the CF lung this variation will influence the apparent abundance of individual bands in T-RFLP profiles. The impact of this phenomenon on T-RFLP profiles has yet to be determined as many bands have no species assignation currently (only ~18% of T-RF band lengths match a band length generated from published sequence data). Moreover, even if all bands were associated with individual species, currently just over one hundred species [24] have been fully sequenced. Iteratively, this will become less problematic as more band-species linkages and genome sequences become available.

In the case of bands not being detected in the corresponding RT-T-RFLP analysis, it is likely that these bacteria were either dead or active at very low metabolic rates. The identification of bands in RT-T-RFLP profiles but absent in the corresponding T-RFLP profile from the same sample therefore suggests that these species are highly active but present in numbers below the threshold of detection. The most interesting such case was the identification of a band corresponding to *B. cepacia *complex, a known CF pathogen, in RT-T-RFLP profiles but not the corresponding T-RFLP profiles. This suggests that *B. cepacia *complex is present in relatively low numbers, but is highly metabolically active. Compared to *P. aeruginosa*, *B. cepacia *complex infects only a small proportion of CF patients, but its impact on survival is significant [25]. Further, the clinical outcome of CF patients colonised by *B. cepacia *complex is much poorer following lung transplantation than their non-infected counterparts [26,27]. For these reasons, the detection of strains of *B. cepacia *complex using these approaches will therefore be carefully monitored in future studies.

The lungs of all individuals are exposed to transient bacteria both that originate both in the oropharyngeal flora and the wider environment. For the detection of such a large number of metabolically active bacterial species in CF sputum to be significant it must be established that they are not due to contamination.

Strenuous attempts were made when processing the sputum samples analysed here to remove bacteria that may have adhered to the sputum bolus during its passage through the upper airways. Further comparisons between the bacterial populations found in CF sputa with those found in mouthwashes obtained from the same patients (data not shown) suggest that there is no significant cross-contamination. Further, here we have analysed the bacterial communities found in sputum obtained from healthy, non-CF individuals. It was found that the majority of the metabolically active bacterial species detected in the CF sputa were not detected at all in non-CF sputa and that bacterial profiles generated from healthy individuals show both a high degree of conservation, and a distinct dissimilarity to those generated from CF sputa. Further, the detection of metabolically active bacterial species in both CF sputum and either CF oropharyngeal community or non-CF sputum is not necessarily an indication of contamination. Bacterial species colonising the CF lung almost certainly derive from either transient bacterial species to which we are all exposed, or to oropharyngeal flora. Therefore, their presence in sputum may well indicate genuine lung colonisation.

Like any other novel approach, data interpretation must be carried out with caution. However, more studies using this and complementary approaches will build upon this characterisation of the complex bacterial community colonising the CF lung. Moreover, this approach can be applied to many other infectious diseases of single or polymicrobial origin. Other diagnostic systems such as gene array applications have clear diagnostic potential [reviewed in [28]]. By their nature however, they can only detect signature sequences for bacteria that have been deposited on the supporting matrix and are, therefore, "closed" systems. The RT-T-RFLP approach that we describe here is an open system where no prior assumptions have been made about the species present in the clinical sample and may, as such, be more flexible. For diagnosis, the RT-T-RFLP system will be developed through the construction of a large database containing digest patterns for individual species. In turn, this will provide clinicians with diagnostic identifications tied to probability value scores. This process translates into more rapid detection – providing results in hours rather than days. This can be equated easily to cost savings in terms of operator time and, where relevant, hospital bed occupancy. Also, there are benefits in terms of more appropriate drug selection. Apart from likely cost savings, this will be particularly important in reducing the impact of long term antibiotic use on patients e.g. renal damage.

The RT-T-RFLP system can offer flexibility in other ways. By switching the PCR primers used, it has the potential to reveal diagnostic information at either the sub-species level, or for fungal or viral infections. Moreover, RT-T-RFLP system can be coupled with Quantitative PCR techniques [29]. With development, RT-T-RFLP has the potential to rapidly assess treatment efficacy by monitoring bacterial viability within clinical samples taken from an individual patient. This monitoring would provide clinicians with data with which to make informed decisions e.g. on dosage modifications or more profound alterations in therapy. Through such studies, this may provide fresh insights into the process of infection in a wide range of respiratory diseases.

## Conclusion

Overall, this study has shown that the majority of bacterial species detected and previously reported in CF lung infections are metabolically active. Further, this suggests that the majority of species detected in samples from the CF lung may play important roles in lung infections. The roles of these bacteria in the pathogenic process occurring in the CF lung are therefore worthy of much further investigation. Moreover, this approach may prove very valuable clinically in the study of many other microbial infections.

## Authors' contributions

All authors participated in the conception and design of the study. GB Rogers carried out all experimental procedures and data analysis. Work was coordinated by KD Bruce. All authors read and approved the final manuscript.

## Supplementary Material

Additional File 1 **Table 2. T-RF band lengths and percentages of total lane signal volume**. The length in bases and the relative proportion of the total lane signal volume (% total) of each of the T-RF bands detected in the T-RFLP and RT-T-RFLP profiles generated from the samples. Click here for file
